# Vaccine targeting to mucosal lymphoid tissues promotes humoral immunity in the gastrointestinal tract

**DOI:** 10.1126/sciadv.adn7786

**Published:** 2024-05-29

**Authors:** Ozgun Kocabiyik, Parastoo Amlashi, A. Lina Vo, Heikyung Suh, Sergio A. Rodriguez-Aponte, Neil C. Dalvie, J. Christopher Love, Raiees Andrabi, Darrell J. Irvine

**Affiliations:** ^1^ Koch Institute for Integrative Cancer Research, Massachusetts Institute of Technology, Cambridge, MA 02139, USA.; ^2^ Consortium for HIV/AIDS Vaccine Development (CHAVD), Scripps Research Institute, La Jolla, CA 92037, USA.; ^3^ Department of Medicine, University of Pennsylvania, Philadelphia, PA 19104, USA.; ^4^ Department of Biological Engineering, Massachusetts Institute of Technology, Cambridge, MA 02139, USA.; ^5^ Department of Chemical Engineering, Massachusetts Institute of Technology, Cambridge, MA 02139, USA.; ^6^ Ragon Institute of Massachusetts General Hospital, Massachusetts Institute of Technology and Harvard University, Cambridge, MA 02139, USA.; ^7^ Department of Materials Science and Engineering, Massachusetts Institute of Technology, Cambridge, MA 02139 USA.; ^8^ Howard Hughes Medical Institute, Chevy Chase, MD 20815 USA.

## Abstract

Viruses, bacteria, and parasites frequently cause infections in the gastrointestinal tract, but traditional vaccination strategies typically elicit little or no mucosal antibody responses. Here, we report a strategy to effectively concentrate immunogens and adjuvants in gut-draining lymph nodes (LNs) to induce gut-associated mucosal immunity. We prepared nanoemulsions (NEs) based on biodegradable oils commonly used as vaccine adjuvants, which encapsulated a potent Toll-like receptor agonist and displayed antigen conjugated to their surface. Following intraperitoneal administration, these NEs accumulated in gut-draining mesenteric LNs, priming strong germinal center responses and promoting B cell class switching to immunoglobulin A (IgA). Optimized NEs elicited 10- to 1000-fold higher antigen-specific IgG and IgA titers in the serum and feces, respectively, compared to free antigen mixed with NE, and strong neutralizing antibody titers against severe acute respiratory syndrome coronavirus 2. Thus, robust gut humoral immunity can be elicited by exploiting the unique lymphatic collection pathways of the gut with a lymph-targeting vaccine formulation.

## Teaser

Intraperitoneal injection of engineered squalene-based vaccines elicits robust mucosal immunity in the gut.

## INTRODUCTION

Many viruses infect the mucosal and lymphoid tissues of the lower gastrointestinal (GI) tract (*1*–*4*). For example, HIV can be transmitted via the rectal mucosa and spreads to adjacent sites (*5*, *6*). Gut-associated lymphoid tissues (GALT) are near this site of viral entry and are rich with CD4^+^ T cells that HIV primarily infects (*7*–*9*). Other viruses, such as severe acute respiratory syndrome coronavirus 2 (SARS-CoV-2) and rotavirus, can directly infect the intestinal mucosa since intestinal epithelial cells express their target receptors (*10*–*12*). Furthermore, recent evidence suggests that persistence of SARS-CoV-2 in intestinal tissues may be a driver of post-acute sequelae of COVID-19 (“long COVID”) (*13*). In addition to viruses, a variety of bacteria and parasites also cause GI infections (*14*–*16*).

To provide optimal protection from such pathogens, mucosal immunity and, particularly, mucosal antibody responses are highly desirable. Secretory immunoglobulin A (IgA) antibodies are optimized for mucosal defense as they have greater stability than monomeric IgA and IgG in the GI mucosa (*17*, *18*). In addition, secretory IgA provides more effective virus neutralization and enhances trapping of antigen in mucus compared to monomeric IgA or IgG in the GI tract (*19*–*22*). However, traditional routes of immunization, such as intramuscular injection, prime robust systemic immunity but typically fail to elicit sustained responses at mucosal portals of pathogen entry (*23*, *24*). Gut mucosal immune responses are programmed in the GALT and mesenteric lymph nodes (mesLNs), but delivery of vaccines to these inductive sites is a major challenge. Antigen uptake directly from the gut lumen can occur via capture by dendritic cells (DCs) residing in the gut epithelium, direct transfer to cognate B cells by M cells overlying Peyer’s patches, or traffic in lymph to the mesLNs (*25*–*27*). However, orally administered vaccines must survive the proteolytic environment of the GI tract, penetrate the mucus overlying the gut epithelium, and overcome the tolerogenic state favored for gut-derived antigens (*28*–*31*). Thus, of five oral vaccines licensed for use in the United States, four are based on live-attenuated pathogens that have evolved to infect the GI tract (*28*). However, use of live-attenuated vaccines is problematic for use against highly mutable pathogens such as HIV.

Nanoparticles naturally clear from tissues by convection into lymphatics rather than entering the blood vasculature (*27*, *32*). Notably, in addition to collecting lymph fluid from the gut, the mesLNs are part of a lymphatic chain that drains the peritoneal cavity (*33*), and nanoparticles administered intraperitoneally have been shown to accumulate in mediastinal LNs and mesLNs (*34*). Although intraperitoneal administration is not traditionally considered for vaccine administration, it is used in the clinic for administration of microencapsulated cell therapies (*35*) and chemotherapy (*36*) and has been used as a route for gene therapy and cancer vaccines in the setting of ovarian cancer (*37*–*39*). We thus hypothesized that intraperitoneal administration of particles carrying both antigen and adjuvant compounds could target vaccines to mesLNs and provide a practical route to induce strong gut-associated mucosal immunity.

To test this idea, we sought to build on clinically safe and effective nanoparticle vaccine adjuvants based on oil-in-water nanoemulsions (NEs). These adjuvants are composed of surfactant-stabilized biodegradable oils and are used in multiple licensed vaccines including Fluad (MF59-adjuvanted influenza vaccine from Novartis) and Pandemrix (AS03-adjuvanted influenza vaccine from GSK) (*40*–*42*). Traditional NE adjuvants have been shown to not directly bind to coformulated antigens (*41*), and thus we designed NEs that could carry an associated protein immunogen by linking antigens to an amphiphilic poly(ethylene glycol) (PEG)–lipid localized at the surface of the NE. To further amplify the immunogenicity of these vaccines, we encapsulated the potent adjuvant Toll-like receptor 7/8 (TLR7/8) agonist 3M-052 (*43*, *44*), which is being used in multiple ongoing vaccine clinical trials, in the oil phase of the NE. We found that these antigen/adjuvant-carrying NEs efficiently targeted mesLNs following intraperitoneal but not subcutaneous immunization. Persistence of antigen in these mucosa-draining LNs could be optimized by anchoring the antigen to a cholesterol tail that allowed exchange of the antigen from the NE to surrounding cells in the lymphoid tissue. Optimized NEs promoted robust germinal center (GC) responses in mesLNs and IgA class switching, leading to strong and sustained serum and mucosal antigen-specific IgA responses. These findings suggest that engineered nanoparticles can be used to efficiently prime gut mucosal immunity by exploiting the unique lymph drainage patterns of the intraperitoneal space.

## RESULTS

### Oil-in-water NEs can serve as dual antigen/adjuvant carriers

**Fig. 1.**
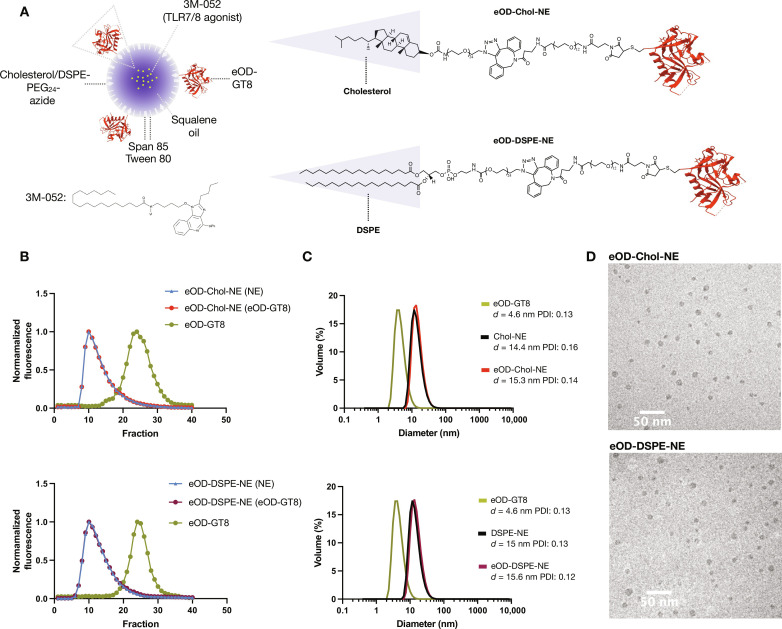
Synthesis and characterization of antigen-coupled NEs. (**A**) Schematic of NE/eOD-GT8 conjugate. The linker is cholesterol-PEG_24_-azide (eOD-Chol-NE) or DSPE-PEG_24_-azide (eOD-DSPE-NE). (**B**) SEC profiles of eOD-Chol-NE and eOD-DSPE-NE together with unconjugated eOD-GT8. SEC experiments were conducted with fluorescent NE carrying BODIPY-cholesteryl ester and AF647-conjugated eOD-GT8. (**C**) DLS analysis (size distribution by volume) of eOD-Chol-NE and eOD-DSPE-NE together with unconjugated eOD-GT8 and NEs. PDI, polydispersity index. (**D**) Representative cryo-TEM images of the eOD-Chol-NE and eOD-DSPE-NE.

We sought to engineer a squalene-based NE (consisting of squalene oil, Span 85, and Tween 80) (*45*, *46*) as a carrier to codeliver antigen and a potent TLR agonist to mucosa-draining lymphoid tissues. We incorporated the TLR7/8 agonist 3M-052 in the emulsion oil phase and anchored protein antigens to the surface of the emulsion droplets by reacting dibenzocyclooctyne (DBCO)–derivatized antigens with azide groups installed at the surface of the emulsion droplets by inclusion of one of two different azide-functionalized PEG-lipid surfactants in the NE: cholesterol-PEG_24_-azide or 1,2-distearoyl-*sn*-glycero-3-phosphoethanolamine-PEG_24_-azide (DSPE-PEG_24_-azide; Fig. 1A). As a testbed immunogen, we selected a germline-targeting antigen termed engineered outer domain–GT8 (eOD-GT8), which was developed to prime B cells against the CD4 binding of HIV Env gp120 (*47*–*49*), and recently shown in a phase 1 clinical trial to successfully prime VRC01-class memory B cells in humans (*50*). Monomeric eOD-GT8 was fused at its C terminus with the pan human leukocyte antigen DR–binding epitope peptide (*51*, *52*) to provide extra T cell help, and a terminal free cysteine was introduced at the N terminus to enable coupling to maleimide-containing molecules. This modified Cys-eOD-GT8 was coupled to DBCO-PEG_12_-maleimide to enable conjugation of the immunogen to the NEs via azide-DBCO cycloaddition (fig. S1, A and B).

Although currently licensed NE adjuvants have mean particle sizes of 100 to 200 nm in diameter (*53*, *54*), we exploited a previously described protocol (*45*) to prepare NEs with a smaller mean size to promote efficient lymphatic delivery. Azide-functionalized PEG-lipid surfactants and 3M-052 were dissolved in dichloromethane and added to a mixture of squalene oil, Span 85, and Tween 80. After evaporating the organic solvent, citrate buffer was added to form NEs by self-assembly in a single step. Incorporation of 3M-052 into the NEs was confirmed by ultraviolet-visible spectroscopy and size exclusion chromatography (SEC) (fig. S2, A to F). DBCO-modified eOD-GT8 was then conjugated to the NEs bearing cholesterol or DSPE-anchored PEG-azide groups to form eOD-GT8/NE conjugates (referred to hereafter as eOD-Chol-NE and eOD-DSPE-NE, respectively; fig. S2G), which were separated from unreacted antigen by centrifugal filtration. Coupling of fluorescently labeled eOD-GT8 antigen to fluorescent NEs followed by SEC analysis showed that the antigen comigrated with the NE after coupling (Fig. 1B). Dynamic light scattering (DLS) showed that Chol-NE and DSPE-NE exhibited a small shift to increased size following antigen coupling, consistent with the small size of the eOD antigen (Fig. 1C and fig. S2H). Cryo–transmission electron microscopy (cryo-TEM) measurements also showed a diameter for both antigen-conjugated NEs in the range of 10 to 15 nm (Fig. 1D). The composition and properties of the NEs are summarized in table S1. As a control, free eOD-GT8 protein was physically mixed with TLR7a-loaded NEs without chemical conjugation (hereafter, eOD/NE).

### NEs target antigen to mucosa-draining lymph nodes following intraperitoneal injection

**Fig. 2.**
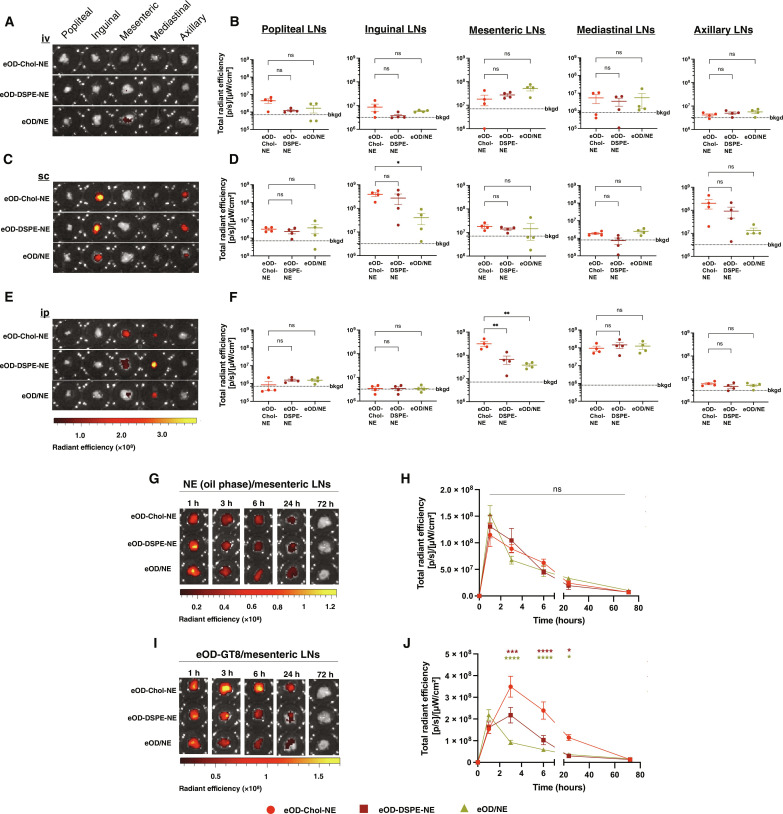
NEs target cholesterol-anchored antigen to mesLNs. (**A** to **F**) Representative IVIS images [(A), (C), and (E)] and quantification [(B), (D), and (F)] of fluorescence from AF647-labeled eOD in excised LNs 6 hours following intravenous (iv) [(A) and (B)], subcutaneous (sc) [(C) and (D)], or intraperitoneal (ip) [(E) and (F)] injection of eOD-Chol-NE, eOD-DSPE-NE, or eOD/NE (10 μg of eOD, 5 μg of 3M-052, and 0.5 μg of squalene NE per vaccine dose, *n* = 4 mice per group). Statistical significance was determined by one-way analysis of variance (ANOVA) followed by Tukey’s post hoc test. (**G** to **J**) Fluorophore-labeled (eOD-AF647 and NE-BODIPY cholesteryl ester) eOD-Chol-NE, eOD-DSPE-NE, and eOD/NE formulations were injected intraperitoneally in BALB/c mice [*n* = 4 animals per group, same dose as (A) to (F)] and at different time points; mesLNs were isolated for IVIS imaging. Shown are representative fluorescence images of NE [oil phase; (G)] and eOD antigen (I) accumulation and mean NE fluorescence signals of NE (H) and eOD antigen (J) over time. Data shown are from one representative of two independent experiments. Statistical significance was determined by two-way ANOVA followed by Tukey’s post hoc test. Not significant, ns; *P* > 0.05; *P < 0.05; **P < 0.01; ***P < 0.001; ****P < 0.0001. All data show means ± SEM. bkgd, background.

We first assessed the in vivo biodistribution behavior of antigen-NE conjugates following different routes of administration. eOD-GT8 antigen was labeled with an Alexa Fluor 647 (AF647) dye prior to NE conjugation, and then eOD-Chol-NE, eOD-DSPE-NE, or eOD/NE formulations were administered to mice intravenously, subcutaneously at the tail base, or intraperitoneally. Six hours after injection, popliteal, inguinal, mesenteric, posterior mediastinal, axillary, and brachial LNs were harvested and whole-tissue antigen uptake was quantified by in vivo imaging system (IVIS) fluorescence measurements (Fig. 2, A to F). Following intravenous administration, very little antigen uptake was detected in all of the LNs analyzed, for all three vaccine formulations (Fig. 2, A and B). Subcutaneous administration led to substantial vaccine targeting to the immediate draining inguinal LNs for the two antigen-conjugated NE formulations but no uptake in the gut-draining mesLNs (Fig. 2, C and D). By contrast, intraperitoneal administration led to antigen accumulation in mesLNs and mediastinal LNs, with NEs carrying cholesterol-anchored antigen providing a significantly increased mesLN uptake of ~5-fold over eOD-DSPE-NE or eOD/NE formulations (Fig. 2, E and F).

Given the promising biodistribution seen for intraperitoneally administered NEs, we next conducted a study to simultaneously characterize the kinetics of antigen and NE accumulation and clearance from mesLNs. BODIPY-cholesteryl ester, a non-exchangeable lipophilic dye (*55*, *56*), was incorporated to track the oil core of the NEs, while the eOD-GT8 antigen was AF647 dye labeled as before. After intraperitoneal administration of fluorophore-modified eOD-Chol-NE, eOD-DSPE-NE, or eOD/NE, mesLNs were harvested at different time points and analyzed by IVIS. The NE signal peaked for all three formulations at ~1 hour after administration and then decreased over time, in a manner indistinguishable for the three groups (Fig. 2, G and H). By contrast, the antigen kinetics for each group were distinct. While eOD-GT8 mixed with NE showed peak accumulation at 1 hour followed by steady clearance, roughly mirroring the pattern of NE kinetics, the eOD-GT8 anchored to NE via cholesterol or DSPE lipids showed peak accumulation later, at ~3 hours (Fig. 2, I and J). Notably, by 24 hours, the antigen was largely cleared from mesLNs for the eOD-DSPE-NE and eOD/NE formulations, while some antigen delivered by the eOD-Chol-NE still persisted at this time point (Fig. 2J). We also conducted a control experiment with eOD-GT8 conjugated to free cholesterol-PEG_24_-azide (i.e., without NE). In this case, eOD-GT8 accumulation in the mesLNs was two to three times lower in comparison to eOD-Chol-NE (fig. S3, A to C). Thus, NEs carrying cholesterol-anchored eOD effectively delivered antigen to the mesLNs.

**Fig. 3.**
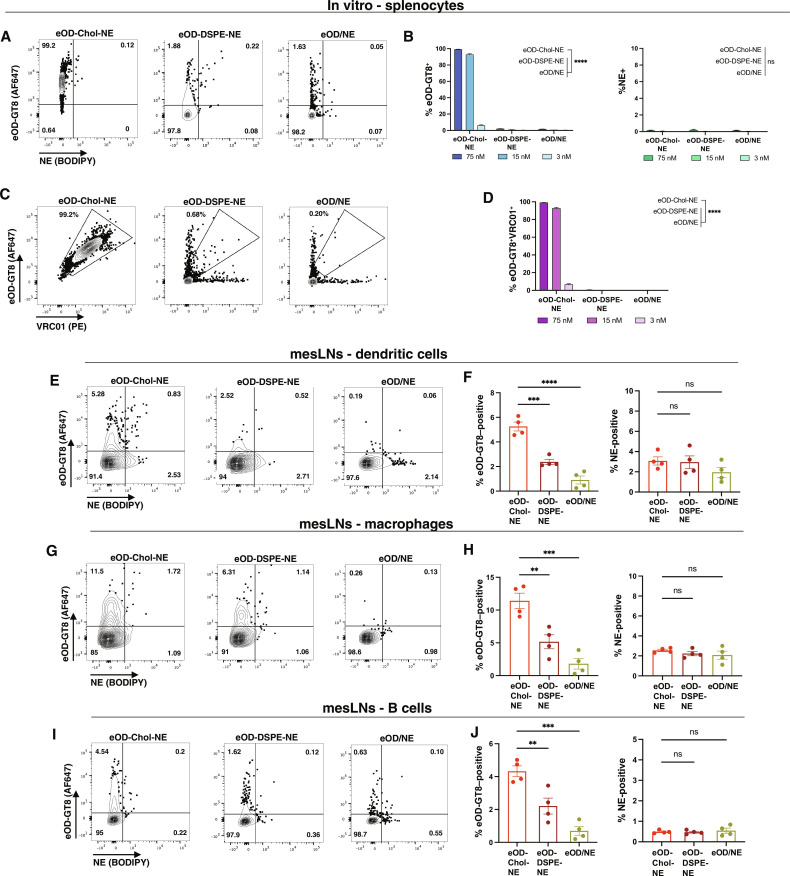
Cholesterol-conjugated antigen transfers from NEs to lymphocyte membranes in vitro and in vivo. (**A** to **D**) Splenocytes were incubated with fluorescently labeled eOD-Chol-NE, eOD-DSPE-NE, or eOD/NE at the indicated concentrations for 1 hour in RPMI medium with 5% fetal bovine serum at 37°C, then washed, and stained with VRC01-PE antibody (*n* = 3 samples per group). Shown are representative flow cytometry plots of eOD-GT8 and NE uptake by splenocytes (at 75 nM eOD-GT8) (A), the percentage of eOD-GT8 (left) or NE-positive cells (right) as a function of eOD-GT8 concentration (B), representative flow cytometry plots of eOD-GT8 signal and VRC01 binding (C), and quantification of the mean frequency of eOD-GT8^+^VRC01^+^ double-positive cells as a function of eOD-GT8 concentration (D). Statistical significance was determined by two-way ANOVA followed by Tukey’s post hoc test. ns, *P* > 0.05; *P < 0.05; **P < 0.01; ***P < 0.001; ****P < 0.0001. (**E** to **J**) BALB/c mice (*n* = 4 mice per group) were injected intraperitoneally with fluorescent eOD-Chol-NE, eOD-DSPE-NE, and eOD/NE (10 μg of eOD, 5 μg of 3M-052, and 0.1 μg of squalene NE), mesLNs were collected 24 hours later, and eOD-GT8/NE uptake by the cells was analyzed by flow cytometry. Shown are representative flow cytometry plots showing eOD-GT8 and NE uptake by DCs (E), macrophages (G), and B cells (I); and quantification of mean frequencies of eOD-GTE8–positive or NE-positive cells [(F), (H), and (J)]. Data shown are from one representative of two independent experiments. Statistical significance was determined by one-way ANOVA followed by Tukey’s post hoc test. ns, *P* > 0.05; *P < 0.05; **P < 0.01; ***P < 0.001; ****P < 0.0001. All data show means ± SEM.

Amphiphilic molecules can spontaneously exchange between organized lipid structures such as emulsions, lipoproteins, extracellular vesicles, and cell membranes, and the chemical structure of the amphiphile plays an important role in the exchange rate (*57*). Given the data above showing more prolonged retention of cholesterol-anchored eOD-GT8 in mesLNs compared to the NE itself, we hypothesized that the cholesterol-anchored antigen might be dissociating from NEs in mesLNs and decorating cell membranes in the tissue, thereby promoting prolonged tissue retention. To explore this idea, we first assessed eOD-GT8 and NE uptake by lymphocytes in vitro. Fluorescent eOD-Chol-NE, eOD-DSPE-NE, or eOD/NE was incubated with splenocytes at different molar concentrations of the antigen in the presence of serum for 1 hour at 37°C, followed by washing and flow cytometry analysis. Splenocytes showed minimal uptake of the NE on this timeframe (Fig. 3, A and B). Notably, however, at higher antigen concentrations, cholesterol-anchored eOD-GT8 was transferred to more than 90% of the cells after incubation with eOD-Chol-NE, while only less than 2% of the cells took up DSPE-anchored eOD-GT8 or free antigen mixed with NE at the same concentrations (Fig. 3, A and B). We hypothesized that this transfer to cells could reflect either cell membrane insertion or endocytosis of the cholesterol-PEG-conjugate; to distinguish these possibilities, we conjugated dye-labeled eOD-GT8 to NEs, incubated with splenocytes for 1 hour, washed, and then stained the cells with the HIV broadly neutralizing antibody VRC01, which binds with high affinity to the eOD-GT8 antigen (*47*, *58*). As shown in Fig. 3 (C and D), cholesterol-anchored eOD-GT8 associated with the cells was essentially all detectable by VRC01, with VRC01 staining proportional to the amount of eOD-GT8 uptake, suggesting predominantly cell surface plasma membrane insertion of the conjugate. This cell surface decoration was cell type–independent as nearly the entire splenocyte population became surface labeled following eOD-Chol-NE incubation. By contrast, negligible amounts of DSPE-anchored eOD-GT8 or free antigen that transferred to the cells could be stained by VRC01 (Fig. 3, C and D). Similar results were obtained when NEs or free eOD-GT8 was incubated with splenocytes in the absence of serum, suggesting that serum proteins did not play a critical role in this cell transfer behavior (fig. S4).

We next sought to determine whether cell transfer of the cholesterol-anchored antigen also occurs in lymphoid tissues in vivo. We injected the same formulations intraperitoneally, and mesLNs were harvested 24 hours later to assess eOD-GT8/NE uptake by antigen-presenting cells and lymphocytes (fig. S5A). In line with the in vitro experiments, eOD-GT8 showed substantially greater association with CD11b^−^CD11c^+^ DCs (Fig. 3, E and F), CD11b^+^F4/80^+^ macrophages (Fig. 3, G and H), and B cells (Fig. 3, I and J) when administered as eOD-Chol-NE compared to the other two vaccine formulations. As observed in vitro, association of lipidated eOD antigen was non–cell type-selective, and we also observed uptake of lipidated eOD-GT8 antigen by T cells (fig. S5, B and C). Cells that were antigen-positive were generally negative for NE uptake, suggesting dissociation of the antigen from the NE in the LNs (Fig. 3, E, G, and I). These data suggest that the effective LN accumulation of cholesterol-anchored antigen reflects a dynamic process of NE targeting to mesLNs and subsequent transfer to LN-resident cell populations.

### NE targeting of antigen and adjuvant to mesLNs primes strong systemic and mucosal immune responses

**Fig. 4.**
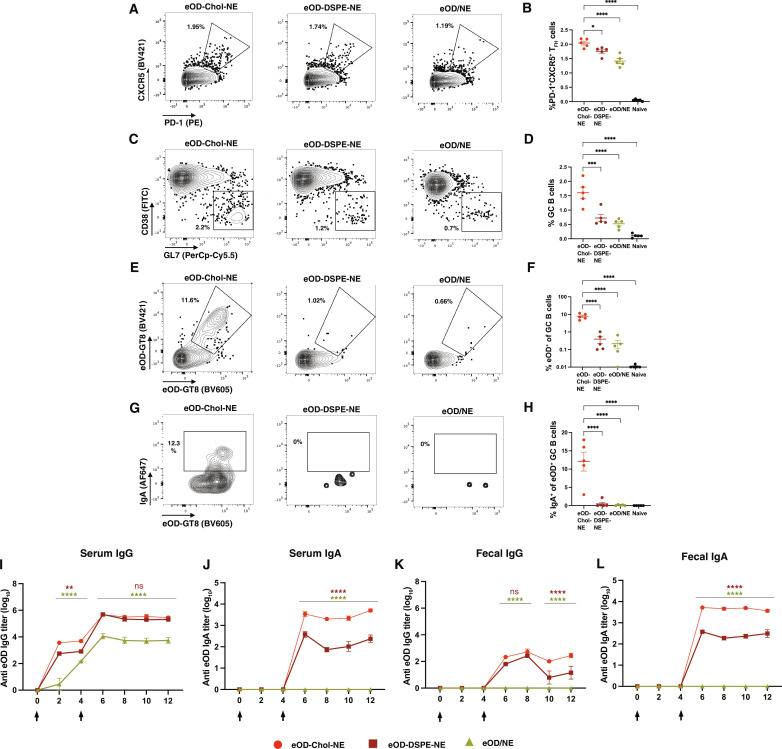
Targeting of vaccines to mesLNs using NEs amplifies mucosal GC and antibody responses. (**A** to **H**) BALB/c mice (*n* = 5 mice per group) were immunized with eOD-Chol-NE, eOD-DSPE-NE, or eoD/NE (10 μg of eOD-GT8, 5 μg of 3M-052, and 0.1 μg of squalene NE), and GC responses in mesLNs were analyzed by flow cytometry at day 12. Shown are representative flow cytometry gating of PD-1^+^CXCR5^+^ T_FH_ cells (A), total GC B cells (C), eOD-GT8–binding GC B cells (E), and IgA^+^ eOD-GT8–binding GC B cells [gated on the (E) gate] (G), as well as mean frequencies of T_FH_ (B), GC B cells (D), eOD-GT8–binding GC B cells (F), and IgA^+^eOD-GT8^+^ GC B cells (H). FITC, fluorescein isothiocyanate. Statistical significance was determined by one-way ANOVA followed by Tukey’s post hoc test. ns, *P* > 0.05; *P < 0.05; **P < 0.01; ***P < 0.001; ****P < 0.0001. (**I** to **L**) BALB/c mice (*n* = 5 animals per group) were immunized with eOD-Chol-NE, eOD-DSPE-NE, or eoD/NE (~10 μg of eOD-GT8, 5 μg of 3M-052, and 0.1 μg of squalene NE) and boosted 4 weeks later, and anti-eOD titers in serum and feces were measured over time. Shown are serum IgG titers (I), serum IgA titers (J), fecal IgG titers (K), and fecal IgA titers (L). Data shown are from one representative of two independent experiments. Statistical significance was determined by two-way ANOVA followed by Tukey’s post hoc test. ns, *P* > 0.05; *P < 0.05; **P < 0.01; ***P < 0.001; ****P < 0.0001. All data show means ± SEM.

To assess immune responses elicited by NEs, we immunized mice with eOD-Chol-NE, eOD-DSPE-NE, or eOD/NE intraperitoneally, and mesLNs were harvested for flow cytometry analysis of GC responses 12 days later (fig. S6). eOD-Chol-NE induced the highest frequency of both PD-1^+^CXCR5^+^ T follicular helper (T_FH_) cells and total GC B cells (Fig. 4, A to D). Most notably, when we stained GC B cells with eOD-GT8 antigen tetramers, the antigen-specific GC B cells population primed by eOD-Chol-NE was ~20-fold greater than the other two formulations (Fig. 4, E and F). Moreover, a portion of these eOD-GT8–specific GC B cells were class switched to IgA, as desired for optimal mucosal immunity (Fig. 4, G and H).

In parallel, we immunized mice with the same formulations, boosted 4 weeks later, and measured Ig titers longitudinally by enzyme-linked immunosorbent assay (ELISA). Unlike free antigen mixed with NE adjuvant, both antigen-conjugated NEs induced systemic IgG in all animals by 2 weeks after prime, and responses to eOD-Chol-NE and eOD-DSPE-NE remained ~100-fold higher than the eOD/NE condition after boost (Fig. 4I and fig. S7). eOD-Chol-NE induced the highest serum IgG titers after the prime, ~5 times higher than eOD-DSPE-NE and ~10 times higher than eOD/NE. Serum IgA was absent in all groups after prime, but following the boost, robust IgA titers were detected for both antigen-conjugated NEs, with the cholesterol-anchored NE eliciting ~10-fold higher titers than the eOD-DSPE-NE group (Fig. 4J). To assess mucosal antibody responses elicited in the gut, fecal Ig titers were also measured. Low fecal IgG titers were detectable after the boost for the antigen-conjugated NEs (Fig. 4K). By contrast, after boost, the antigen-coupled NEs elicited strong fecal IgA titers that were stable through at least 12 weeks, and similar to the serum data, eOD-Chol-NE elicited responses ~15-fold greater than eOD-DSPE-NE (Fig. 4L). As expected, control subcutaneous administration of eOD-Chol-NE induced high systemic antibody titers but no mucosal IgA (fig. S8). Notably, immunization using the eOD-Chol-NE formulation without encapsulated 3M-052 elicited much weaker GC responses and no IgA class switching in the mesLNs (fig. S9, A to C) and failed to prime systemic or mucosal IgA, indicating the importance of the codelivered TLR agonist (fig. S9, D to G).

**Fig. 5.**
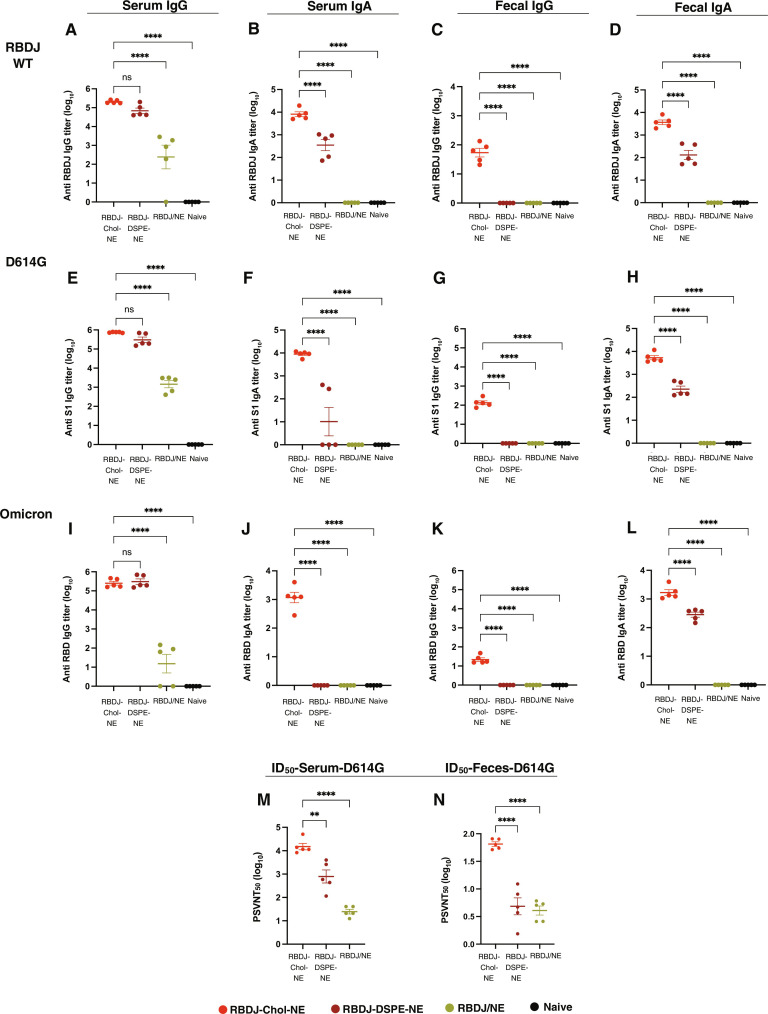
NE RBD vaccines promote cross-reactive SARS-CoV-2 systemic and gut mucosal antibody responses. BALB/c mice (*n* = 5 mice per group) were immunized with RBDJ-Chol-NE, RBDJ-DSPE-NE, or RBDJ/NE (10 μg of RBD, 5 μg of 3M-052, and 0.1 μg of squalene NE) and boosted 4 weeks later. Shown are serum IgG titers (**A**, **E**, and **I**), serum IgA titers (**B**, **F**, and **J**), fecal IgG titers (**C**, **G**, and **K**), and fecal IgA titers (**D**, **H**, and **L**) at week 6 against RBDJ, the S1 protein of the SARS-CoV-2 D614G variant, and RBD protein of the SARS-CoV-2 Omicron variant, respectively. WT, wild type. (**M** and **N**) Serum (M) and feces (N) SARS-CoV-2 D614G variant pseudovirus neutralizing ID_50_ titers (PSVNT_50_). Statistical significance was determined by one-way ANOVA followed by Tukey’s post hoc test. ns, *P* > 0.05; *P < 0.05; **P < 0.01; ***P < 0.001; ****P < 0.0001. All data show means ± SEM.

eOD-GT8 is clinically relevant as a germ line–targeting antigen for HIV VRC01-class broadly neutralizing antibodies in humans, but wild-type mice cannot generate VRC01-class neutralizing antibody responses. Thus, to test the capacity of mesLN-targeted vaccination to promote protective gut antibody responses, we carried out immunizations with a second antigen, an engineered mutant of the SARS-CoV-2 receptor binding domain (RBD) termed RBDJ (RBD-L452K-F490W) based on the Wuhan SARS-CoV-2 sequence (*59*, *60*). NEs were prepared with equivalent compositions as used for the eOD-GT8 immunogen (table S1), and mice were primed and boosted with RBD-carrying NEs. We assessed antibody titers in the serum and feces of the mice against the WT RBDJ as well as two different SARS-CoV-2 variant antigens: whole S1 spike protein from the D614G variant and RBD from Omicron. Mirroring the findings with eOD-GT8, RBDJ-Chol-NE, and RBDJ-DSPE-NE elicited serum IgG responses that were two orders of magnitude higher than free RBDJ mixed with NEs, and these sera also recognized the D614G and Omicron variants (Fig. 5, A, E, and I). RBDJ-Chol-NE also elicited strong serum and fecal IgA titers against all three antigens (Fig. 5, B, F, J, D, H, and L). As with eOD-GT8, RBDJ mixed with NE was unable to prime any mucosal Ig responses. Although RBDJ-Chol-NE induced high serum and fecal IgA titers, we did not detect IgA titers in the bronchoalveolar lavage (BAL) fluid (BALF) (fig. S10). However, high BALF IgG titers were measured for both RBDJ-DSPE-NE and RBDJ-Chol-NE immunizations.

The protective capacity of these antibody responses was assessed by a pseudovirus neutralizing assay. RBDJ-Chol-NE immunization elicited a serum neutralizing ID_50_ (50% inhibitory dose) titer of ~10^4^ against SARS-CoV-2 D614G, ~10 and ~300 times higher than RBDJ-DSPE-NE and RBDJ/NE immunizations, respectively (Fig. 5M). Moreover, RBDJ-Chol-NE immunization elicited readily detectable fecal neutralizing ID_50_ titers, ~10-fold greater than RBDJ-DSPE-NE and RBDJ/NE immunizations (Fig. 5N). These data suggest that targeting mesLNs with NE vaccines is an effective strategy to promote neutralizing antibody responses in the gut mucosa.

## DISCUSSION

Induction of mucosal antibody responses, especially IgA, in the GI tract is desirable for optimal protection from a variety of pathogens that infect through the gut epithelium, but achieving robust gut immunity using nonlive microbial vaccines has been challenging. Most commonly, vaccines aiming to promote gut immunity are administered by the oral route (*28*, *61*, *62*), and antigens that penetrate the gut mucus are captured from the intestinal lumen by M cells in the small intestine and transferred to antigen-presenting cells in the Peyer’s patches. In addition, DCs dispersed among epithelial cells of the gut can extend processes into the gut lumen to sample antigen (*25*) and migrate to lymphoid follicles and mesLNs where B cell class switching to IgA-producing cells is induced (*63*, *64*). However, oral vaccination is limited by the efficiency of these antigen uptake processes and the tendency of gut antigen uptake to promote tolerance rather than immunity (*28*, *29*). Here, we exploited the fact that mesLNs collect lymph not only from the gut but also from the peritoneal space and targeted vaccines to this gut immunity inductive site via a simple intraperitoneal injection using vaccine-loaded NEs. Formulation of vaccines in particulate carriers is a well-established approach to limit antigen/adjuvant dispersal into the bloodstream from tissues and instead favor lymphatic uptake and to additionally promote vaccine capture at draining LNs (*27*, *32*). To favor the safety of this approach, we used NEs based on the biodegradable oil squalene used in several licensed vaccines and a TLR agonist that has been shown to be safe in ongoing clinical trials.

We conjugated antigen to these NE vaccines via PEG-lipids, motivated by the desire to mimic viral display of many copies of antigen at the particle surfaces. As the chemical structure of lipids is known to influence their tendency to exchange with serum proteins/membranes (*65*, *66*), we tested two different lipid “anchors”: DSPE and cholesterol. Unexpectedly, NEs carrying antigen linked via a cholesterol anchor were substantially more effective in achieving antigen accumulation in mesLNs and drove much stronger GC and gut antibody responses than the less exchangeable DSPE lipid tail. These enhanced vaccine responses correlated with an ability of the cholesterol-anchored antigen to transfer from the NE to lymphocytes in the mesLNs, which we suspect is favored due to the dense cellularity of LNs compared to the intraperitoneal space.

In addition to strong mucosal IgA, NE vaccines also stimulated strong systemic antibody responses. Serum IgG titers and neutralizing responses to the SARS-CoV-2 antigen delivered by NEs intraperitoneally were comparable to published data in mice for mRNA vaccines (*67*, *68*), although we expect these mRNA vaccines administered intramuscularly would elicit little or no mucosal response. DSPE-anchored antigen NEs also worked well for priming serum IgG, and this was true for both the eOD-GT8 and COVID antigens. We speculate that class switching to IgA requires more prolonged antigen exposure than switching to IgG and that this is only achieved by the cholesterol-conjugated antigen that transfers to cells in the mesLNs. By contrast, we suspect that class switching for systemic IgG can be achieved using both the cholesterol-conjugated emulsion and the more limited antigen persistence achieved by the DSPE formulation, and this may occur in both mesLNs and mediastinal LNs.

Vaccine administration via intramuscular injection is often the preferred route to administer vaccines due to the low reactogenicity of intramuscularly dosed vaccines (*69*), and intraperitoneal administration in humans is generally avoided due to the risk of misplaced injections. However, these risks can be mitigated by techniques such as ultrasound-guided injection (*70*). Administering tetanus toxoid (TT) or measles virus vaccines via the intraperitoneal route has been shown to be well tolerated in humans (*38*, *71*). Notably, the trial immunizing against TT using a traditional TT vaccine failed to elicit mucosal IgA in humans (*71*), consistent with our preclinical findings that unformulated antigens are not efficiently delivered to the mucosa-draining lymphoid tissues. This route is also used in the clinic for administration of cell therapies (*35*), chemotherapy (*36*), gene therapy, and cancer vaccines in the setting of ovarian cancer (*37*–*39*). Thus, while more complex than a simple intramuscular injection, the potential of intraperitoneal vaccination to promote strong humoral immunity in the gut (and other mucosal tissues) is an important advantage for gut-tropic pathogens. Recent approaches to increase the safety and efficacy of peritoneal catheter insertion, such as ultrasound-guided peritoneal puncture (*72*), may provide a means to enable this approach for mass vaccination.

In conclusion, this study demonstrates the potential of intraperitoneal injection of engineered squalene-based vaccines to elicit robust mucosal immunity in the gut. This approach holds promise for enhancing protection against infectious diseases in the lower GI tract, particularly when oral vaccine delivery is not feasible or effective.

## MATERIALS AND METHODS

### NE/immunogen conjugate preparation

The TLR7 agonist 3M-052 (0.5 mg; Cayman Chemical) and 1 mg of cholesterol-PEG_24_-azide or DSPE-PEG_24_-azide (BroadPharm) was dissolved in 100 μl of dichloromethane and mixed with 10 μl of squalene oil (Sigma-Aldrich), 5 μl of Span 85 (Sigma-Aldrich), and 37.5 mg of TWEEN 80 (Sigma-Aldrich). To prepare fluorescent NEs, 0.25 mg of CholEsteryl BODIPY (Thermo Fisher Scientific) dissolved in 100 μl of dichloromethane was also added. The organic solvent was removed using a rotary evaporator, and then 1 ml of 10 mM citrate buffer (pH 6.0, heated to 37°C) was added to form NEs and sonicated for 1 min in an ultrasonic bath (VWR B1500A-DTH; 50 W at 42 kHz). The solution was filtered with 0.1-μm syringe filters and characterized by DLS and TEM.

HIV env gp120 eOD-GT8 and SARS-CoV-2 spike RBD protein antigens with N-terminal cysteines were prepared as previously reported (*60*, *73*). Briefly, Cys-modified antigens at a concentration of 1 and 0.5 mg/ml, respectively, were reduced with 4 molar equivalents of tris(2-carboxyethyl)phosphine for 15 min at 25°C in phosphate-buffered saline (PBS). The reduced proteins (1 mg/ml) were then reacted with 10 equivalents DBCO-PEG_12_-maleimide (Sigma-Aldrich) in PBS for 18 hours at 25°C. Unreacted linker was removed from the samples by centrifugal filtration using 10-kDa molecular weight cutoff (MWCO) Amicon spin filters. Free protein controls were not modified with DBCO-PEG_12_-maleimide to prevent conjugation to the NEs. To prepare fluorescent proteins, 10 equivalents of AF647-NHS ester dye (Thermo Fisher Scientific) was mixed with proteins and left on an orbital shaker for 18 hours at 25°C. Unconjugated dye was removed by centrifugal filtration using 10-kDa MWCO Amicon spin filters.

To form NE/immunogen conjugates, ~55 μl of the NE was added to 350 μl of PBS and centrifuged twice using 50-kDa MWCO Amicon spin filters for buffer exchange into PBS. The resulting NEs and 80 μg of modified protein were mixed and reacted on an orbital shaker for 18 hours at 25°C. Unbound protein was removed by centrifugal filtration using 100-kDa MWCO Amicon spin filters and characterized by SEC.

### Animals

Experiments and handling of the mice were conducted in accordance with the National Institutes of Health (NIH) ethical guidelines, following an Institutional Animal Care and Use Committee (IACUC)–approved protocol. Female BALB/c mice (6 to 8 weeks old) were purchased from the Jackson Laboratory (stock no. 000651).

### In vivo biodistribution studies with IVIS

In vivo trafficking of the eOD-Chol-NE, eOD-DSPE-NE, and eOD/NE was assessed following intraperitoneal administration using IVIS fluorescence imaging (PerkinElmer). BALB/c mice were immunized with formulations containing 10 μg of AF647-eOD-GT8, 5 μg of CholEsteryl BODIPY 542/563 C_11_, 5 μg of 3M-052, and 0.1 μg of squalene oil. After 1, 3, 6, 24, or 72 hours, mesLNs and other LNs as indicated were harvested. AF647 and BODIPY fluorescence (radiant efficiency) was measured on freshly excised tissues.

### eOD-GT8/NE cellular uptake studies in vitro

NE/immunogen uptake by cells was assessed in vitro using murine splenocytes as previously reported (*60*). Fluorescently labeled eOD-Chol-NE, eOD-DSPE-NE, and eOD/NE formulations (eOD-GT8 modified with AF647, NEs prepared with encapsulated CholEsteryl BODIPY FL C_12_, and 3M-052) were incubated with 2 × 10^6^ cells/ml (4 × 10^5^ cells per well in a 96-well plate) in complete RPMI medium [RPMI 1640 + 5% fetal bovine serum (FBS)] or in FBS-free RPMI medium at different dilutions: 3, 15, or 75 nM eOD-GT8. Following 1-hour incubation, cells were washed with PBS, stained with Zombie Aqua (BioLegend) at 1:1000 dilution in 100 μl of PBS for 15 min at 25°C, washed with flow cytometry buffer [PBS + 2% bovine serum albumin (BSA)], and then stained with the Env CD4 binding site–specific monoclonal antibody PE-VRC01 at 1 μg per 10^6^ cells in 100 μl of flow cytometry buffer for 20 min at 25°C. Cells were then washed twice and fixed using 2% paraformaldehyde (PFA). Data acquisition was performed using BD LSR II and analyzed with FlowJo 10.

### eOD-GT8/NE cellular uptake studies in vivo

In vivo uptake studies were carried out following procedures we previously developed (*60*): BALB/c mice were immunized with NE formulations containing 10 μg of AF647-eOD-GT8, 5 μg of green fluorescent BODIPY FL C_12_ cholesteryl ester, 5 μg of 3M-052, and 0.1 μg of squalene oil. Twenty-four hours later, mesLNs were collected and single-cell suspensions were prepared. Cells were first incubated with live/dead stain (Zombie UV, BioLegend) at 1:750 dilution in 100 μl of PBS for 15 min at 25°C and then with antimouse CD16/32 antibody (TruStain FcX, BioLegend) at 1:100 dilution in 50 μl of flow cytometry buffer for 10 min at 25°C. Cells were stained against antimouse CD3ε PE-CF594 (clone 145-2C11; BD Biosciences), CD19 BUV395 (1D3; BD Biosciences), CD11b BUV737 (M1/70; BD Biosciences), CD11c BV711 (N418, BioLegend), F4/80 PE (BM8; BioLegend), Ly6C BV785 (HK1.4, BioLegend), and Ly6G BV421 (1A8, BioLegend) at 1:100 dilution in 100 μl of flow cytometry buffer for 15 min at 25°C. Cells were washed twice and fixed using 2% PFA. Data acquisition was performed using BD FACSymphony A3 and analyzed with FlowJo 10.

### T_FH_ cell response

T_FH_ responses were analyzed as previously reported (*60*): BALB/c mice were immunized with NE formulations containing 10 μg of eOD-GT8, 5 μg of 3M-052, and 0.1 μg of squalene oil. After 12 days, mesLNs were collected and single-cell suspensions were prepared. Cells were first incubated with live/dead stain (Zombie Aqua, BioLegend) at 1:750 dilution in 100 μl of PBS for 10 min at 25°C and then with antimouse CD16/32 antibody at 1:100 dilution in 100 μl of flow cytometry buffer for 10 min at 25°C. Cells were stained against antimouse CD19 FITC at 1:200 (clone 1D3; BioLegend), CD4 AF647 at 1:200 (GK1.5; BioLegend), CD44 PE-Cy7 at 1:200 (IM7; BioLegend), PD-1 PE at 1:50 (RMP1-14; BioLegend), and CXCR5 BV421 at 1:50 (L138D7; BioLegend) in 100 μl of flow cytometry buffer for 20 min at 25°C. Cells were washed twice and fixed using 2% PFA. Data acquisition was performed using BD LSR II and analyzed with FlowJo 10.

### GC B cell response

GC responses were analyzed as previously reported (*60*): BALB/c mice were immunized with NE formulations containing 10 μg of eOD-GT8, 5 μg of 3M-052, and 0.1 μg of squalene oil. After 12 days, mesLNs were collected and single-cell suspensions were prepared. Cells were first incubated with live/dead stain (Zombie UV, BioLegend) at 1:750 dilution in 100 μl of PBS for 10 min at 25°C and then with antimouse CD16/32 antibody at 1:100 dilution in 100 μl of fluorescence-activated cell sorting buffer for 10 min at 25°C. Cells were stained against antimouse CD90.2 BV785 at 1:200 (clone 30-H12; BioLegend), CD19 BUV395 at 1:200 (1D3; BD Biosciences), CD38 FITC at 1:200 (90; BioLegend), GL7 PerCP-Cy5.5 at 1:150 (GL7; BioLegend), IgA AF647 at 1:100 (SouthernBiotech), eOD-tetramer BV605 at 1:100, and eOD-tetramer BV421 at 1:100 [tetramers formed by incubating 4 equivalents of biotinylated eOD with 1 equivalent of streptavidin-BV605 or streptavidin-BV421 (BioLegend) 18 hours at 4°C, prior to staining] in 100 μl of flow cytometry buffer for 20 min at 25°C. Cells were washed twice and fixed using 2% PFA. Data acquisition was performed using BD FACSymphony A3 and analyzed with FlowJo 10.

### Mouse sample collection

Fecal sample preparation and BALF collection were performed as previously described (*60*). Fecal samples were prepared from mouse fecal pellets. Two pellets of ~0.75 cm each per mouse were added to 200 μl of PBS supplemented with cOmplete protease inhibitor cocktail (11836153001, Roche), and samples were vortexed, kept overnight at 4°C, vortexed again, and then centrifuged at 12,000*g* for 5 min to collect the supernatant. BALF was collected from instillations of PBS (2 × 1 ml) supplemented with cOmplete protease inhibitor cocktail (Roche) in the lungs using a catheter through the trachea. Samples were centrifuged at 12,000*g* for 5 min to collect the supernatant.

### ELISA analysis of antibody titers

ELISA analysis was carried out as previously reported (*60*): To determine eOD-specific or RBD-specific IgA and IgG titers, Costar Polystyrene High Binding 96-well plates (Corning) were coated with eOD antigen or RBD antigen at 2 μg/ml in PBS overnight at 4°C and blocked with blocking buffer (PBS + 2% BSA) for 2 hours at 25°C. Mouse serum samples were diluted in blocking buffer starting at 1:50 followed by 4× serial dilutions, and fecal samples were diluted in blocking buffer starting at 1:50 followed by 3× serial dilutions. Samples were incubated in plates for 2 hours at 25°C. To determine antigen-specific IgG antibodies, goat antimouse IgG–horseradish peroxidase (HRP; 1:5000; BioRad) was added to the wells and incubated for 1 hour. To determine antigen-specific IgA antibodies, goat antimouse IgA-biotin (1:5000; SouthernBiotech) was added and incubated for 1 hour followed by incubation with streptavidin-HRP (1:40,000; Thermo Fisher Scientific Pierce High Sensitivity Streptavidin-HRP) for 30 min. Plates were developed using a 3,3′,5,5′-tetramethylbenzidine substrate for 1 to 5 min and stopped using 2 M sulfuric acid, and the absorbance (450 nm with 540-nm reference) was measured on a plate reader. Endpoint cutoff titers are reported as inverse serum dilutions giving an HRP absorbance of 0.3 above background.

### SARS-CoV-2 pseudovirus neutralization assay

The SARS-CoV-2 pseudoviruses (D614G variant) expressing green fluorescent protein (GFP) were purchased from GeneCopoeia. Twenty-four hours before the experiment, human embryonic kidney (HEK) 293T expressing human ACE2 (hACE2) (GeneCopoeia) were plated in 96-well tissue culture plates at a density of 1 × 10^4^ cells per well. Before running the neutralization assay, serum and fecal samples were heat-inactivated at 56°C for 30 min. Serum samples were diluted in Dulbecco’s modified Eagle’s medium (+10% FBS and p/s) at 1:50 followed by 5× serial dilutions, and fecal samples were diluted at 1:10 followed by 5× serial dilutions. Diluted samples were mixed with pseudovirus (~10^5^ relative light units per well) and incubated at 37°C for 1 hour before adding to HEK293T-hACE2 cells. Polybrene (5 mg/ml) was also added to the wells. At 24 hours, the old medium was replaced with fresh complete medium. Forty-eight hours after, cells were analyzed by flow cytometry. Pseudovirus neutralization titers were determined as the dilution of the sample at which there was a 50% decrease in GFP-positive cells compared to the average of the virus control wells.

### Statistics

Statistics were analyzed using GraphPad Prism software. Statistical comparison was performed using a one-way ANOVA followed by Tukey’s post hoc test for single–time point data and two-way ANOVA followed by Tukey’s post hoc test for multi–time point longitudinal data.
