# Supplementary material for: Vaccine targeting to mucosal lymphoid tissues promotes humoral immunity in the gastrointestinal tract
Source: Sci Adv. 2024 May 29;10(22):eadn7786. doi: 10.1126/sciadv.adn7786 (PMC11135404; doi:10.1126/sciadv.adn7786)
Supplement: Supplementary file 1 — Figs. S1 to S10 Table S1 Legend for data S1 [file sciadv.adn7786_sm.pdf]

Science Advances
AAAS

Supplementary Materials for

Vaccine targeting to mucosal lymphoid tissues promotes humoral immunity in the gastrointestinal tract

Ozgun Kocabiyik *et al.*

Corresponding author: Darrell J. Irvine, djirvine@mit.edu

Sci. Adv. **10**, eadn7786 (2024)
DOI: 10.1126/sciadv.adn7786

The PDF file includes:

Figs. S1 to S10
Table S1
Legend for data S1

Other Supplementary Material for this manuscript includes the following:

Data S1

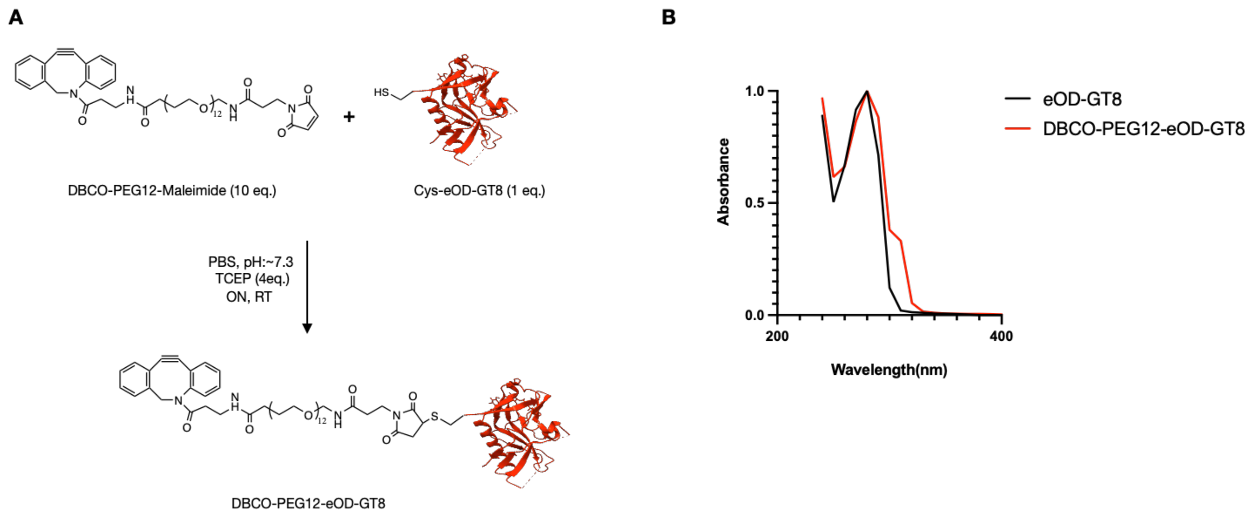

Fig. S1. Synthesis and characterization of DBCO-PEG12-Maleimide modified eOD-GT8.
(A) A schematic of eOD-GT8 conjugation protocol to DBCO-PEG12-Maleimide. (**B**) and absorbance curve before and after the conjugation.

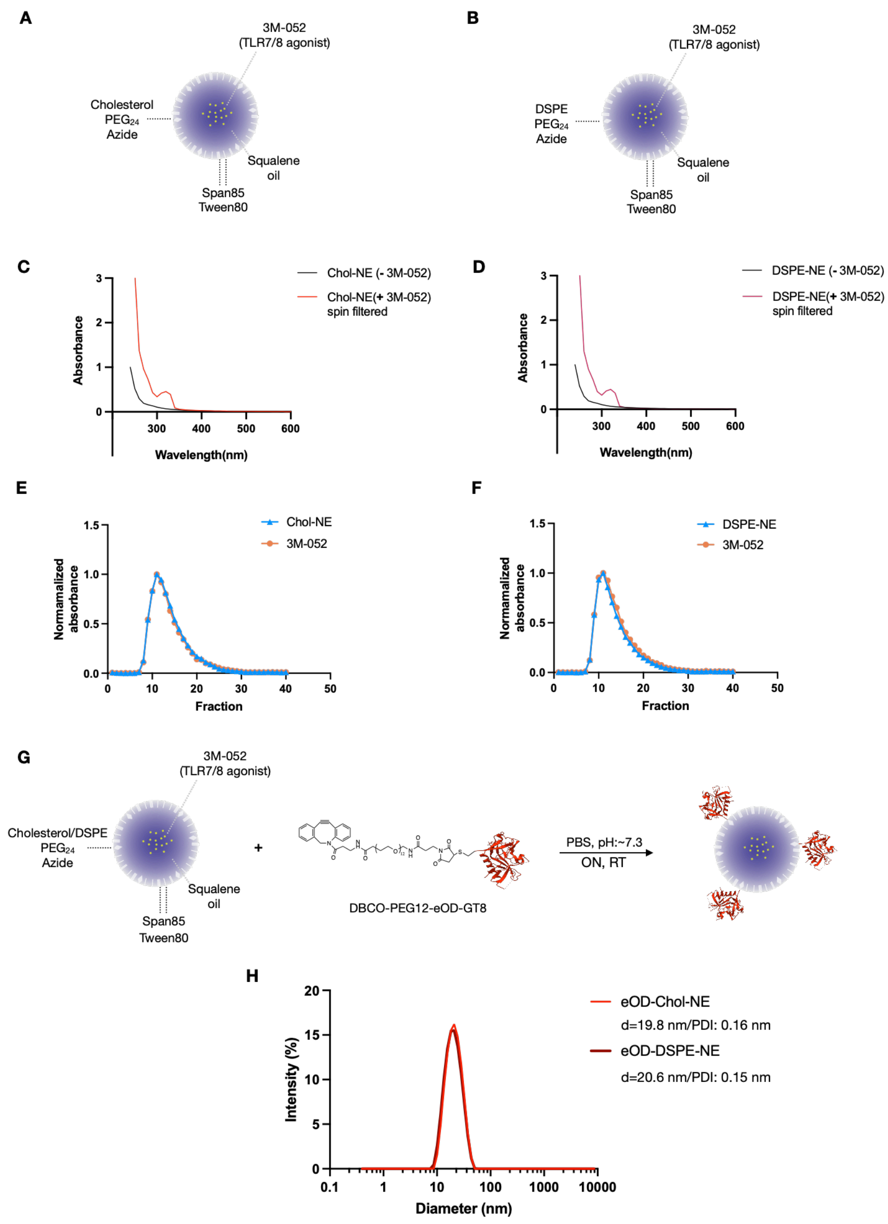

Fig. S2. Synthesis and characterization of antigen conjugated NEs.

(**A** and **B**) Schematic of 3M-052-loaded Cholesterol-PEG$_{24}$-Azide (Chol-NE, A) or DSPE-PEG$_{24}$-Azide (DSPE-NE, B). (**C** and **D**) Absorbance curves of the Chol-NE (C) and DSPE-NE (D) with and without 3M-052. The peak at ~320 nm is specific to 3M-052. (**E** and **F**) Size exclusion chromatography (SEC) profiles of Chol-NE (E) and DSPE-NE (F) showing 3M-052 incorporation in the NEs. For SEC analysis, BODIPY-cholesteryl ester was encapsulated in the NEs and absorbance wavelength of the dye (at 510 nm) was used to trace the NEs together with the absorbance wavelength of 3M-052. (**G**) A schematic of DBCO modified eOD-GT8 conjugation to the NEs. (**H**) DLS analysis (size distribution by intensity) of eOD-Chol-NE and eOD-DSPE-NE.

	Squalene oil	Tween80	Span85	Cholesterol-PEG24-Azide	3M-052	eOD-GT8	Number of antigens/ NE	Diameter by DLS (nm)/ PDI (volume average)	Zeta potential (mV)
eOD-Chol-NE	85 µg (200 nmol)	375 µg (280 nmol)	47.5 µg (50 nmol)	10 µg (6 nmol)	~5 µg (8.5 nmol)	~10 µg (0.3 nmol)	~3	15.3/0.14	-0.82
	Squalene oil	Tween80	Span85	DSPE-PEG24-Azide	3M-052	eOD-GT8			
eOD-DSPE-NE	85 µg (200 nmol)	375 µg (280 nmol)	47.5 µg (50 nmol)	12 µg (6 nmol)	~5 µg (8.5 nmol)	~10 µg (0.3 nmol)	~3	15.6/0.12	-2.02
	Squalene oil	Tween80	Span85	Cholesterol-PEG24-Azide	3M-052	RBDJ			
RBDJ-Chol-NE	85 µg (200 nmol)	375 µg (280 nmol)	47.5 µg (50 nmol)	10 µg (6 nmol)	~5 µg (8.5 nmol)	~10 µg (0.3 nmol)	~4	16.5/0.15	-1.34
	Squalene oil	Tween80	Span85	DSPE-PEG24-Azide	3M-052	RBDJ			
RBDJ-DSPE-NE	85 µg (200 nmol)	375 µg (280 nmol)	47.5 µg (50 nmol)	12 µg (6 nmol)	~5 µg (8.5 nmol)	~10 µg (0.5 nmol)	~4	17.1/0.12	-2.73

Table S1. Nanoemulsion formulations.

Formulations of different nanoemulsion-immunogen conjugates shown in the paper.

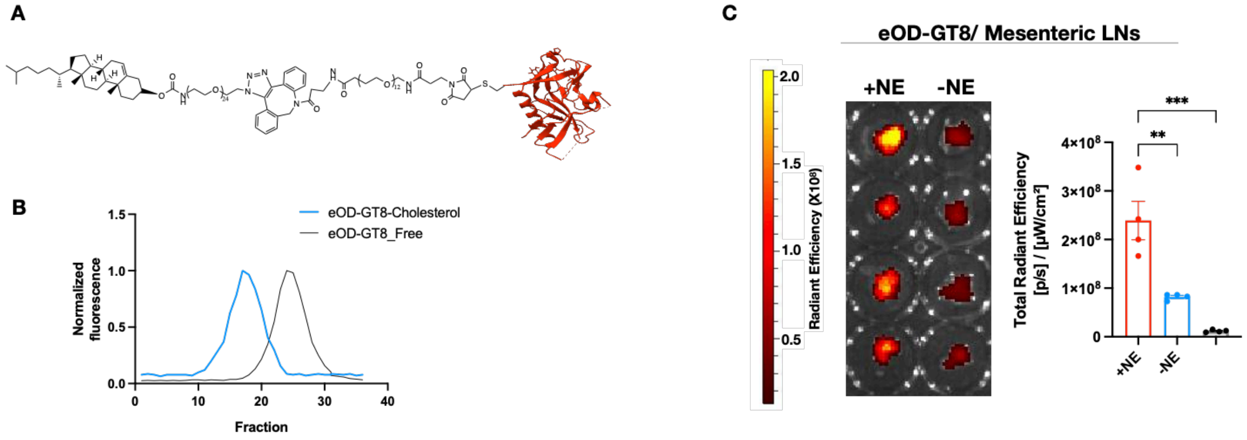

Fig. S3. Schematic, SEC profile and in-vivo trafficking of the eOD-GT8 with cholesterol tail.

(**A**) A schematic of eOD-GT8 conjugated to cholesterol-PEG$_{24}$-azide and (**B**) size exclusion chromatography (SEC) profiles of the conjugate and free eOD-GT8. (**C**) Fluorophore-labeled conjugate and eOD-Chol-NE injected i.p. and 6h later tissues were collected. Representative IVIS images showing eOD-GT8 accumulation in the mesenteric lymph nodes and respective fluorescence quantification. Statistical significance was determined by one-way ANOVA followed by Tukey's post hoc test. $^{**}P < 0.01$; $^{***}P < 0.001$. All data show means ± SEM.

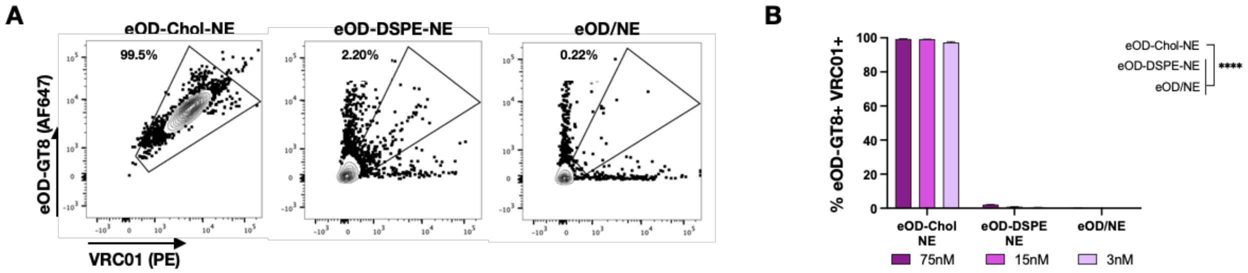

Fig. S4. In-vitro antigen transfer from NEs to the splenocytes in the FBS-free RPMI medium.

Splenocytes were incubated with eOD-Chol-NE, eOD-DSPE-NE or eOD/NE for 1 hour, in FBS-free medium at 37°C at a range of concentrations, then washed and stained with VRC01-PE antibody. (**A**) Representative flow cytometry plots are shown of eOD-GT8 uptake in FBS free RPMI medium and VRC01 binding to the cells (at 75nM of eOD-GT8) (**B**) The percentage of eOD-GT8 and VRC01 positive cells were quantified as a function of eOD-GT8 concentration. Statistical significance was determined by two-way ANOVA followed by Tukey's post hoc test. ****$P < 0.0001$. All data show means ± SEM.

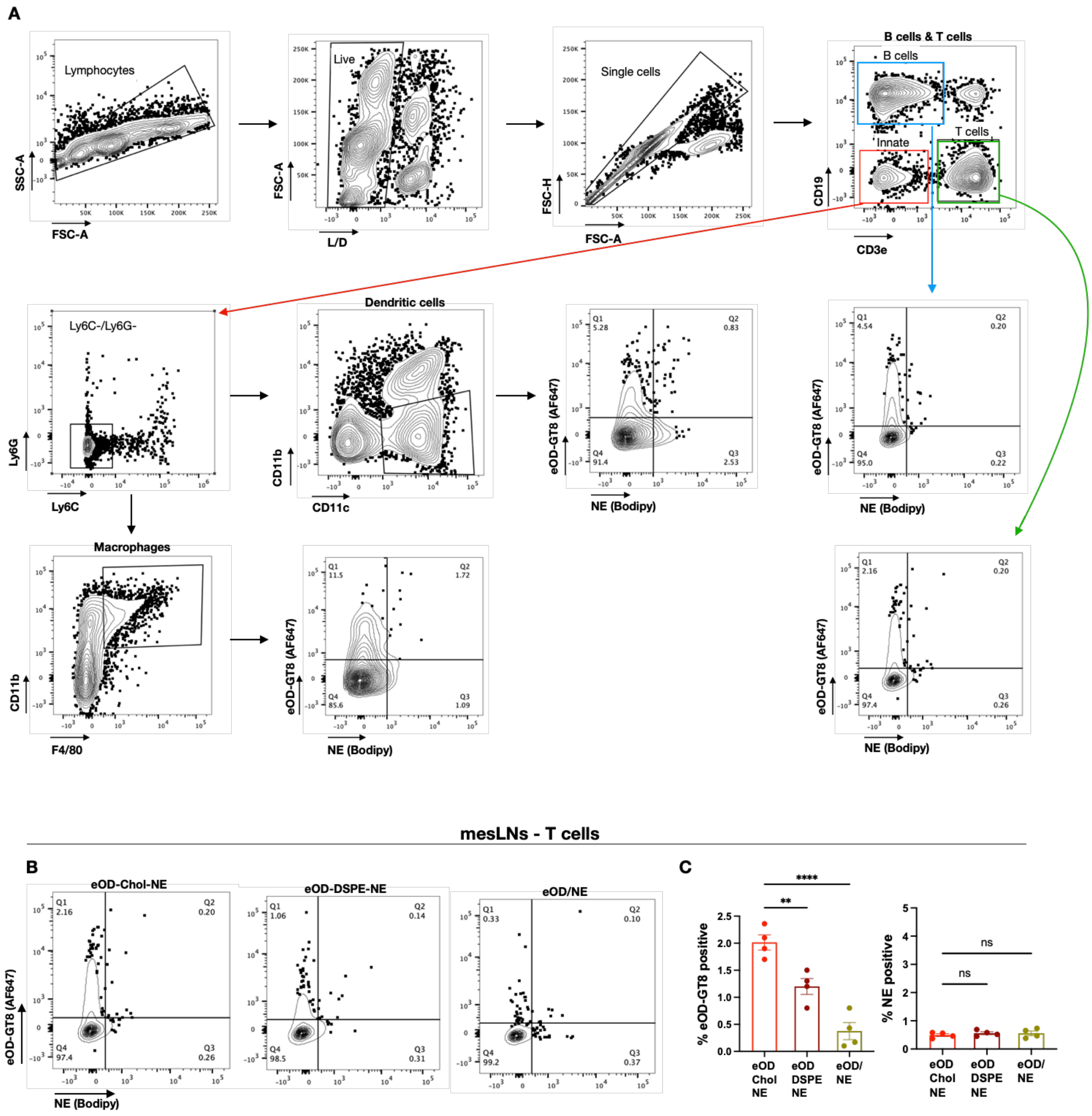

Fig. S5. Flow cytometry gating strategy for eOD-GT8/NE uptake by lymphocytes in mesenteric LNs (A) and eOD-GT8/NE uptake by T cells (B and C)

(A) Mice were immunized with Fluorescent eOD-Chol-NE, eOD-DSPE-NE or eOD/NE. Mesenteric lymph nodes were harvested 24h later for flow cytometry analysis of antigen uptake. Schematic shows gating strategy to identify AF647-eOD-GT8 and NE uptake in B cells, T cells, dendritic cells and macrophages. **(B)** Shown are representative flow cytometry plots showing eOD-GT8 and NE uptake by T cells and **(C)** quantification of mean frequencies of eOD or NE-positive cells. Statistical significance was determined by one-way ANOVA followed by Tukey's post hoc test. ns, $P > 0.05$; $^{**}P < 0.01$; $^{****}P < 0.0001$. All data show means ± SEM.

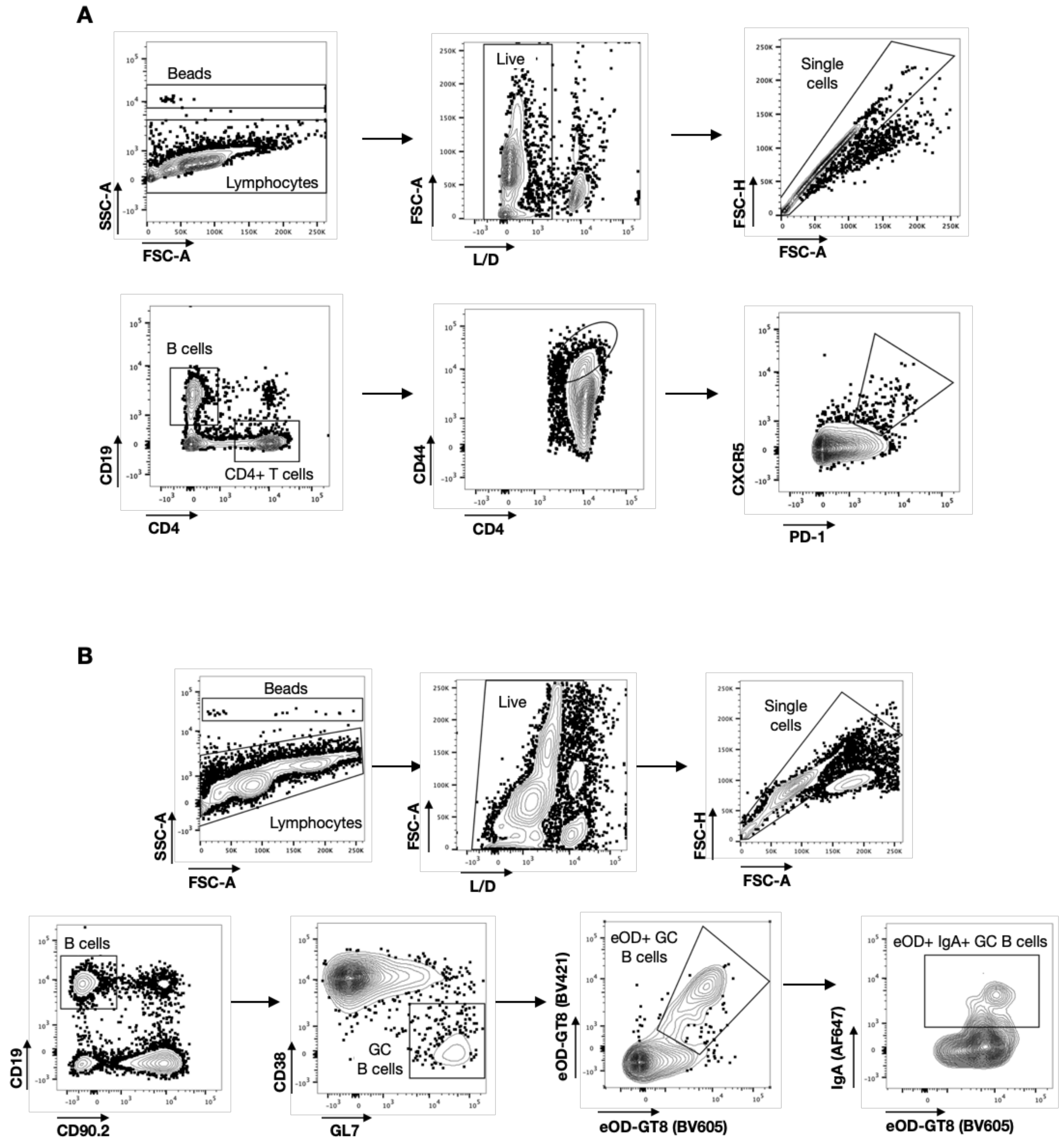

Fig. S6. Flow cytometry gating strategy for GC B cells and Tfh cells.
Mice (n = 5 per group) were immunized with eOD-Chol-NE, eOD-DSPE-NE or eOD/NE. 12 days after, Tfh (**A**) and GC B cell (**B**) responses in mesenteric LNs were analyzed by flow cytometry. Representative gating strategy is shown.

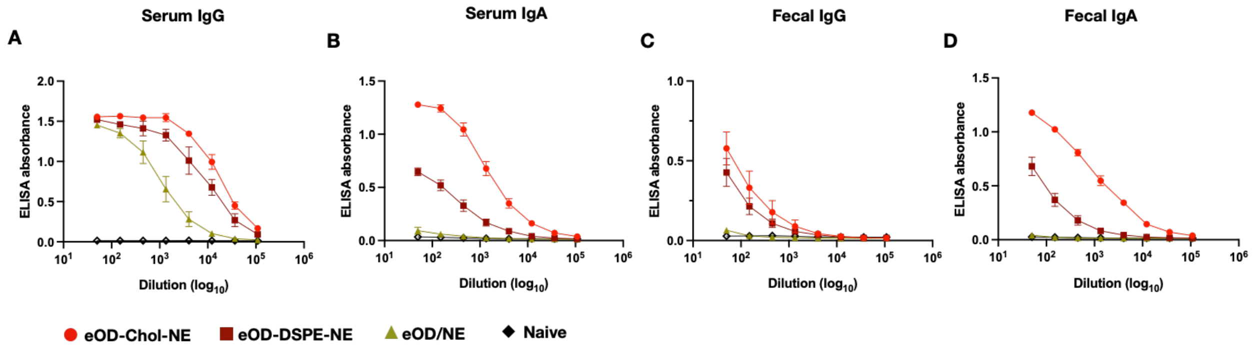

Fig. S7. ELISA absorbance curves from Figure 4.
Mice (n = 5 per group) were immunized with eOD-Chol-NE, eOD-DSPE-NE or eOD/NE and boosted 4 weeks later. Shown are ELISA absorbance vs dilution curves at week 6 for serum IgG (**A**), serum IgA (**B**), fecal IgG (**C**) and fecal IgA (**D**). All data show means ± SEM.

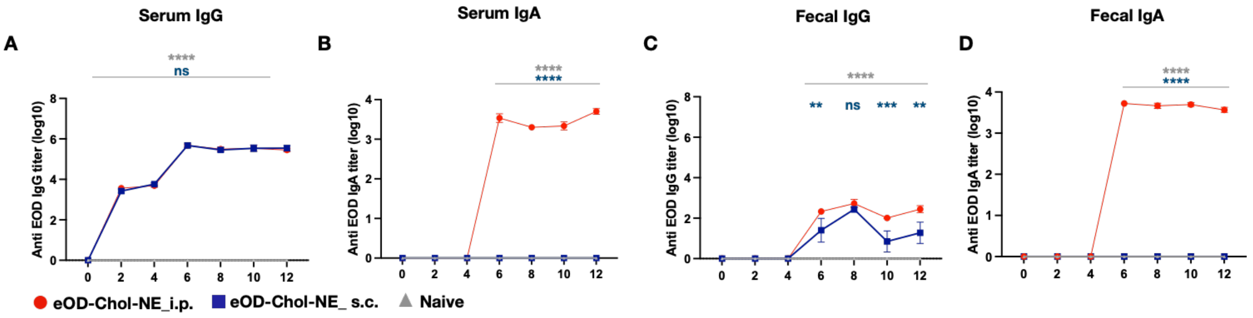

Fig. S8. Comparison of i.p. and s.c. immunization routes.
Mice (n = 5 per group) were immunized with eOD-Chol-NE and boosted 4 weeks later by i.p. or s.c. routes. Shown are serum IgG (**A**) and IgA (**B**) titers, fecal IgG (**C**) and IgA (**D**) titers. Statistical significance was determined by two-way ANOVA followed by Tukey's post hoc test. ns, $P > 0.05$; *$P < 0.05$; **$P < 0.01$; ***$P < 0.001$; ****$P < 0.0001$. All data show means ± SEM.

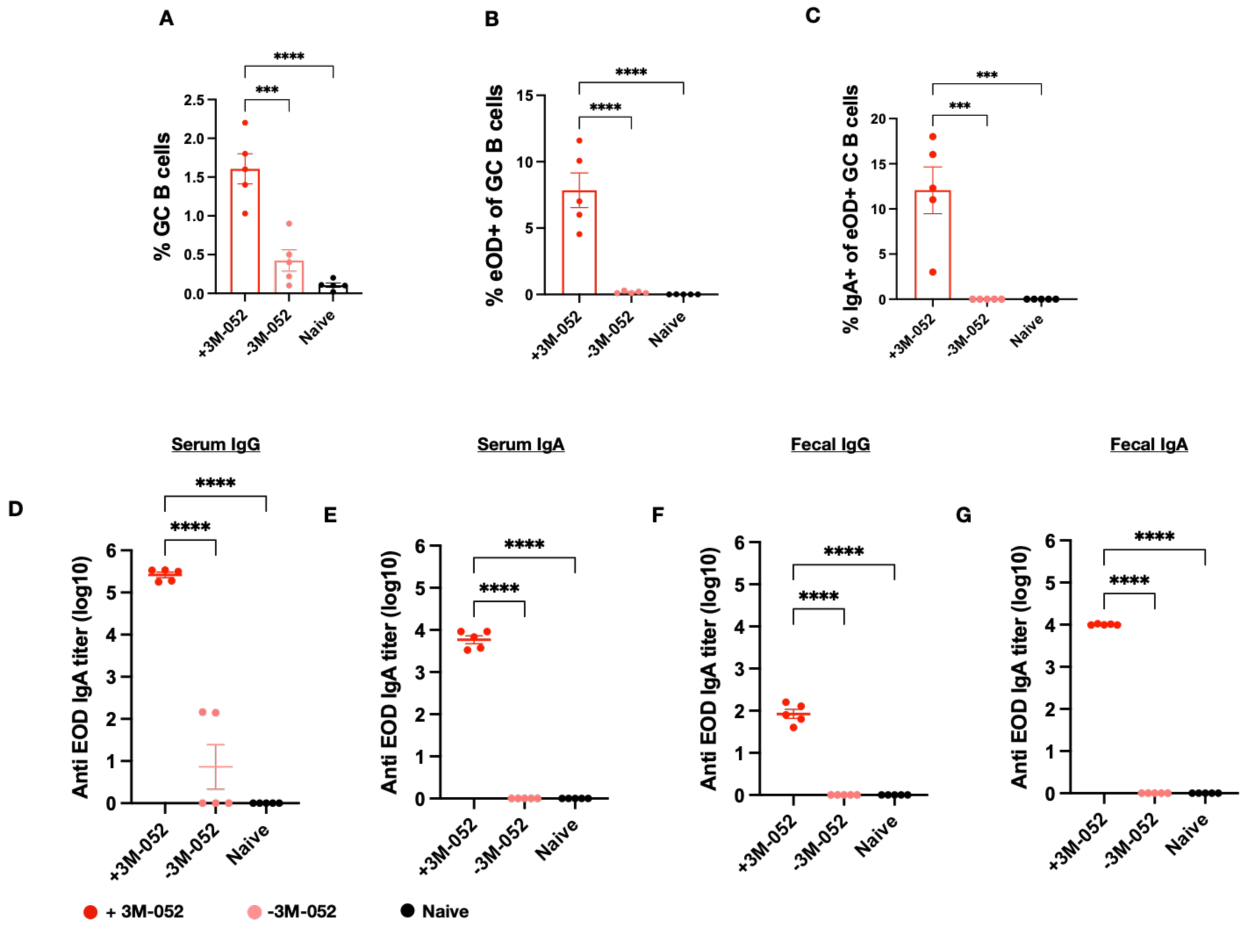

Fig. S9. Comparison of eOD-Chol-NE immunizations with and without 3M-052.
(**A** to **C**) Mice (n = 5 per group) were immunized with eOD-Chol-NE with or without 3M-052. Shown are percentage of GC B cells (A), eOD-GT8+ GC B cells (B), IgA+ eOD-GT8+ GC B cells (C) at day 12. (**D** to **G**) Mice (n = 5 per group) were immunized with eOD-Chol-NE with or without 3M-052 and boosted 4 weeks later. Shown are serum IgG (D) and IgA (E) titers, fecal IgG (F) and IgA (G) titers. at week 6. Statistical significance was determined by one-way ANOVA followed by Tukey's post hoc test. ***$P < 0.001$; ****$P < 0.0001$. All data show means ± SEM.

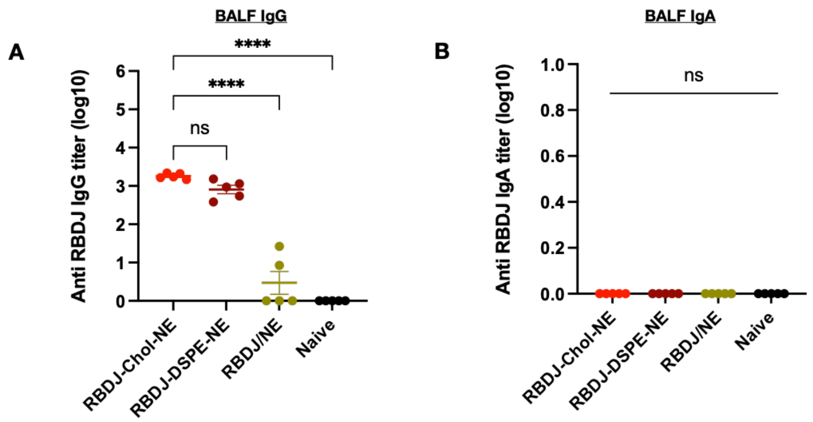

Fig. S10. **BAL fluid (BALf) titers following the RBDJ immunizations.**
Mice (n = 5 per group) were immunized with RBDJ-Chol-NE, RBDJ-DSPE-NE or RBDJ/NE and boosted 4 weeks later. Shown are (BALF IgG (**A**) and IgA (**B**) titers at week 8 against WT RBDJ. All data show means ± SEM.

Data S1. Tabulated individual-level data for main manuscript figures and supplementary figures.
